# Early Changes in Near-Infrared Spectroscopy Are Associated With Cardiac Arrest in Children With Congenital Heart Disease

**DOI:** 10.3389/fped.2022.894125

**Published:** 2022-06-27

**Authors:** Priscilla Yu, Ivie Esangbedo, Xilong Li, Joshua Wolovits, Ravi Thiagarajan, Lakshmi Raman

**Affiliations:** ^1^Division of Critical Care, Department of Pediatrics, University of Texas Southwestern Medical Center, Dallas, TX, United States; ^2^Division of Cardiac Critical Care, Department of Pediatrics, University of Washington Seattle, Seattle, WA, United States; ^3^Department of Population and Data Sciences, University of Texas Southwestern Medical Center, Dallas, TX, United States; ^4^Division of Cardiovascular Critical Care, Department of Pediatrics, Harvard University, Boston, MA, United States

**Keywords:** near-infrared (NIR) spectroscopy, cardiac arrest, prediction, children, congenital heart disease

## Abstract

**Background:**

The association of near-infrared spectroscopy (NIRS) with various outcomes after pediatric cardiac surgery has been studied extensively. However, the role of NIRS in the prediction of cardiac arrest (CA) in children with heart disease has yet to be evaluated. We sought to determine if a model utilizing regional cerebral oximetry (rSO2c) and somatic oximetry (rSO2s) could predict CA in children admitted to a single-center pediatric cardiac intensive care unit (CICU).

**Methods:**

We retrospectively reviewed 160 index CA events for patients admitted to our pediatric CICU between November 2010 and January 2019. We selected 711 control patients who did not have a cardiac arrest. Hourly data was collected from the electronic health record (EHR). We previously created a machine-learning algorithm to predict the risk of CA using EHR data. Univariable analysis was done on these variables, which we then used to create a multivariable logistic regression model. The outputs from the model were presented by odds ratio (OR) and 95% confidence interval (CI).

**Results:**

We created a multivariable model to evaluate the association of CA using five variables: arterial saturation (SpO2)- rSO2c difference, SpO2-rSO2s difference, heart rate, diastolic blood pressure, and vasoactive inotrope score. While the SpO2-rSO2c difference was not a significant contributor to the multivariable model, the SpO2-rSO2s difference was. The average SpO2-rSO2s difference cutoff with the best prognostic accuracy for CA was 29% [CI 26–31%]. In the multivariable model, a 10% increase in the SpO2-rSO2s difference was independently associated with increased odds of CA [OR 1.40 (1.18, 1.67), *P* < 0.001] at 1 h before CA. Our model predicted CA with an AUROC of 0.83 at 1 h before CA.

**Conclusion:**

In this single-center case-control study of children admitted to a pediatric CICU, we created a multivariable model utilizing hourly data from the EHR to predict CA. At 1 h before the event, for every 10% increase in the SpO2-rSO2s difference, the odds of cardiac arrest increased by 40%. These findings are important as the field explores ways to capitalize on the wealth of data at our disposal to improve patient care.

## Introduction

Near-infrared spectroscopy (NIRS) is a method of non-invasive real-time continuous monitoring of tissue oxygenation. The use of NIRS has been studied extensively in children with congenital heart disease (CHD) (1). There have been studies looking at the association between NIRS and various outcomes after pediatric cardiac surgery, such as low cardiac output syndrome, acute kidney injury, necrotizing enterocolitis, neurodevelopmental outcome, and mortality (2–5). In particular, multisite NIRS, which refers to simultaneously monitoring cerebral oxygen saturation (rSO2c) as well as somatic oxygen saturation (rSO2s), can be performed by placing NIRS sensors over the brain and kidney, liver, or intestines. Since the body's response to decreased cardiac output is to decrease perfusion to somatic sites to preserve perfusion to the brain, it can be helpful to monitor multisite NIRS as an early predictor (1). Monitoring regional tissue oxygen extraction (using arterial oxygen saturation-rSO2 difference) can also be useful in the pre- and post-operative periods of children with CHD. In a study of children with CHD during the pre-operative period, it has been shown that those lesions with diastolic runoff compared to lesions without runoff have the most cerebral oxygen extraction indicating less cerebral blood flow. There is also evidence that cerebral tissue oxygen extraction can be predictive of mortality in the children with HLHS after Stage 1 Palliation (6, 7). While the ability of NIRS to predict various outcomes has been well-studied, the role of NIRS in the prediction of cardiac arrest (CA) in children with heart disease has yet to be studied. In fact, this gap in the literature does not only apply to children with CHD. There is literature looking at the association of NIRS with intra-arrest and post-cardiac arrest outcomes in adults (8, 9), although this consists of case reports and case series (10–15). There are no data on the use of NIRS to predict cardiac arrest in adults. Our goal therefore was to determine if regional, cerebral, and somatic oxygen saturations (rSO2c and rSO2s) could predict cardiac arrest in children admitted to a single-center pediatric cardiac intensive care unit (CICU).

## Methods

The Institutional Review Board's approval was obtained before this study. We queried our local Get With the Guidelines Resuscitation (GWTGR) Registry database for children admitted to the Children's Medical Center Dallas pediatric CICU between November 2010 and January 2019 who developed a cardiac arrest during their CICU admission. Our pediatric CICU is a 26-bed unit that admits surgical and non-surgical patients with heart disease. The GWTGR Registry database defines cardiac arrest as pulselessness or pulse with inadequate perfusion requiring chest compressions and/or defibrillation. From the GWTGR Registry database, we retrospectively reviewed all events by chart review of the electronic health record (EHR) to confirm the diagnosis of cardiac arrest. For our study, we chose to only include patients who developed a cardiac arrest that resulted in chest compressions. We excluded cardiac arrests that resulted in defibrillation alone without chest compressions. We chose to analyze only the index events. We identified 160 index CA events during the study period. We attempted to find a method to match controls to cases. Although there exist multiple severities of illness scoring systems that have been validated in the general pediatric ICU population to predict mortality, such as Pediatric Risk of Mortality (PRISM)-3 and Pediatric Index of Mortality (PIM)2, unfortunately, no scoring system has been validated for the population of children with congenital heart disease (16, 17). Jeffries et al. published a scoring tool to predict the risk of mortality, the Pediatric Index of Cardiac Surgical Intensive Care Mortality (PICSIM), that has been validated in children after cardiac surgery (18). Unfortunately, this score is unique to the post-surgical population and cannot be used in non-surgical patients. As there was no validated tool to match cases and controls in total, we selected our control patients at random using the following criteria: 1. Patients admitted to the CICU between November 2009 and December 2019; 2. patients who did not have a cardiac arrest during the admission; 3. patients who were not in the cardiac arrest dataset; and 4. patients who were not on extracorporeal membrane oxygenation (ECMO) already at the time of admission; and 5. patients who were admitted to the CICU within 12 h of hospital admission.

NIRS monitoring was done using Medtronic INVOS oximeter probes and monitors. Our standard of care for all patients who are admitted to our CICU is to place INVOS oximeter probes on the forehead and flank to measure regional cerebral and somatic (kidney) oxygen saturation. We collected various data from the electronic health record (EHR). The study period consisted of up to 48 h before CA for patients who had a cardiac arrest, and up to the first 48 h of ICU admission for the control patients. Demographic data (age, weight, gestational age, ventricular status) were collected from the EHR. Single ventricle physiology is defined as those patients with a mixture of systemic venous and pulmonary venous return, with total cardiac output partitioned into pulmonary and systemic blood flow (19). Therefore, this definition would include patients who are intended to undergo a single ventricle palliation pathway as well as patients palliated initially with a shunt before two ventricle repairs (such as Tetralogy of Fallot). Surgical information (date of surgery, STS-European Association for Cardiothoracic Surgery (STS-EACTS) mortality category (STAT category), cardiopulmonary bypass (CPB) times, and cross-clamp (Xclamp) times were collected from the STS database and EHR).

We had previously created a machine-learning algorithm to predict the risk of CA in our center (manuscript currently under preparation). The machine-learning algorithm was created using XG-boost, or extreme gradient boosting, a type of decision tree algorithm. The machine-learning algorithm was trained on the same dataset. A total of 11 variables were selected that were most important to the machine learning algorithm (Table 1). VIS was defined by the equation: Dopamine dose (mcg/kg/min) + Dobutamine dose (mcg/kg/min) + [100 × Epinephrine dose (mcg/kg/min)] + [10 × Milrinone dose (mcg/kg/min)] + [10,000 × Vasopressin dose (units/kg/min)] + [100 × Norepinephrine dose (mcg/kg/min)]. The data values of HR, SpO2, DBP, rSO2c, rSO2s, and ETCO2 levels are automatically carried over from the patient monitor to the EHR with the bedside nurse confirming the value before it is finalized in the EHR. The missing values were carried forward from the last documented value. The 11 variables in order from most to least missing data were the following: anion gap, FiO2, base excess, ETCO2, rSO2s, rSO1c, DBP, SpO2, HR, urine output, and VIS (Supplementary Table 1). The average of all available values within each hourly interval from hours 1 to 15 before CA was used for analysis. Univariable analysis was performed on each of those 11 variables, and we selected the variables that were found to have an association with CA in our multivariable analysis. We created multivariable logistic regression models to include these variables to predict CA as a function of time. The output from models was presented by odds ratio (OR) and 95% confidence interval (CI).

**Table 1:** Clinical features most important to the XG-boost algorithm.

Heart rate (HR)
Oxygen saturation level measured by pulse oximetry (SpO2)
Diastolic blood pressure (DBP)
rSO2c
rSO2s
End tidal carbon dioxide (ETCO2)
Urine output (cc/kg/hr)
Base excess
Anion gap
Fraction of inspired oxygen (FiO2)
Vasoactive inotrope score (VIS)

For some variables, we used absolute values, while for others, we used the relative change from baseline (Table 2). We defined a patient's baseline for a given variable as the average value over the first 4 h of the study period. The relative change of a given variable X at hour Y was defined as [X (at hour Y)-X (baseline)]/X(baseline. Given the population of children admitted to our CICU have both cyanotic and non-cyanotic heart disease, which can affect the baseline SpO2, rSO2c, and rSO2s, we chose to analyze the changes in these values compared to the patient's baseline rather than their absolute values. In patients with single ventricle physiology, changes in rSO2 can influence the SpO2. Similarly, since baseline HR and DBP can be age-dependent, we elected to analyze changes in HR and DBP compared to the patient's baseline. We elected to use absolute VIS. Values for VIS were not normally distributed therefore could not be represented as a continuous variable in our logistic regression model but rather as categories. When we analyzed the distribution of the VIS data, 75% of the VIS values were 0. Therefore, we elected to classify values for VIS into two categories: VIS 0 and VIS > 0. We also elected to evaluate absolute values of the surrogates of arteriovenous difference: SpO2-rSO2s difference and SpO2-rSO2c difference. We attempted to use simultaneous values of SpO2 and rSO2s and rSO2c to calculate these differences, however, the number of values containing simultaneous values was too small to do an analysis. Therefore, we instead used the average of SpO2 values within a specific hour and the average of rSO2s and rSO2c for that specific hour to calculate the SpO2-rSO2s difference and SpO2-rSO2c difference. All other variables (FiO2, urine output, base excess, anion gap, ETCO2) were represented as absolute values rather than standardized to the patient's baseline. FiO2 was represented as a categorical instead of a continuous variable with all values broken down into tertiles. All values for a given variable over a given hour were averaged and the average value was used in the model. We defined the patient's baseline as the first 4 h of the study period, we excluded any patient who had <6 h of available data. The original contributions presented in the study are publicly available. These data can be found here [link/accession number]. Odds for CA were examined by univariable and multivariable logistic regression models for hours 1 through 15 before CA.

**Table 2:** Clinical features: absolute value vs. relative change from baseline.

**Variable**	**Absolute value or relative change**
Heart rate (HR)	Relative change
Oxygen saturation level measured by	
Pulse oximetry (SpO2)	Relative change
Diastolic blood pressure (DBP)	Relative change
rSO2c	Relative change
rSO2s	Relative change
End tidal carbon dioxide (ETCO2)	Absolute value
Urine output (cc/kg/hr)	Absolute value
Base excess	Absolute value
Anion gap	Absolute value
Fraction of inspired oxygen (FiO2)	Absolute value
Vasoactive inotrope score (VIS)	Absolute value

### Statistical Analysis

Categorical data are presented as counts and proportions and compared between groups using a chi-square test. Mean and std, or Medians and interquartile ranges are used to summarize continuous data and are compared with the *t*-test or Wilcoxon's rank-sum test, based on the distribution of data. Data distribution was assessed by the Shapiro–Wilk's normality test and normal probability plots. Optimal predicted probability cutoffs were determined by Youden's index from the receiver-operating characteristic (ROC) analysis and displayed as median and 95% confidence intervals. All analyses were performed using SAS 9.4 (SAS Institute, Cary, NC). All statistical tests were two-sided, and *P* < 0.05 was considered as significant.

## Results

During the defined study period 160 patients had cardiac arrest and 711 control patients. Table 3 shows the demographics of those patients who had a cardiac arrest vs. no cardiac arrest. Patients who had a cardiac arrest tended to be younger, of lower weight, younger gestational age at birth, more likely to have single ventricle physiology, higher STAT category, and longer CPB and cross-clamp times. The dataset for case and control groups and their corresponding 11 variables for hours 1 to 15 is provided.

**Table 3:** Patient prearrest characteristics: cardiac arrest vs. no cardiac arrest.

**Characteristic**	**Cardiac arrest *n* = 160 (%)**	**No cardiac arrest *N* = 711 (%)**	***P*-value**
Gender (male)	84 (52.50%)	367 (51.62%)	0.84
Age (day) Median [IQR]	54 [1, 908]	217 [30, 1,563]	<0.0001
Number of days from admission to cardiac arrest	10.90 [2.38, 32.80]	N/A	N/A
Single ventricle physiology	64 (40.0)	91 (12.80)	<0.0001
Weight (kg) Median [IQR]	5.17 [3.30, 11.0]	7.30 [4.32, 15.65]	0.0001
Gestational age Median [IQR]	38.0 [36.0, 39.3]	39.0 [37.0, 40.0]	0.0009
For surgical patients: (below variable were analyzed on surgical yes patients only)	*N* = 94	*N* = 337	
Number of post-operative days at arrest Median [IQR]	10.16 [1.68, 26.63]	N/A	
Stat category 1	4 (4.35%)	145 (43.6%)	<0.0001
Stat category 2	17 (18.48%)	109 (32.83%)	
Stat category 3	7 (7.61%)	33 (9.94%)	
Stat category 4	46 (50.00%)	37 (11.14%)	
Stat category 5	16 (17.39%)	5 (1.51%)	
No stat category assigned (stat category = 0)	2 (2.17%)	3 (0.90%)	
CPB times Median [IQR]	118.0 [93.0, 163.0]	77.0 [53.0, 111.0]	<0.0001
X clamp times Median [IQR]	70.0 [39.0, 116.0]	49.5 [24.0, 80.0]	0.003

### Univariable Analysis

Univariable analysis of the different variables with their association with risk of cardiac arrest at 1 h before CA is displayed in Table 4. FiO2, DBP, anion gap, HR, VIS, SpO2, SpO2-rSO2c difference, and SpO2-rSO2s difference were all significantly associated with the risk of cardiac arrest on univariable analysis.

**Table 4:** Univariate predictors of cardiac arrest.

**Variable**	**OR, CI {95%}**	***P*-value**	**AUROC**
FiO2 (tertile 3 vs. 1)	0.78 (0.41, 1.51)	0.47	0.63
(tertile 3 vs. 2)	2.63 (1.59, 4.37)	<0.001	
Urine output	0.98 (0.96, 1.01)	0.30	0.48
DBP	0.91 (0.87, 0.95)	<0.001	0.62
Anion gap	11.20 (3.30, 38.30)	<0.001	0.68
Base excess	0.84 (0.51, 1.40)	0.51	0.51
ETCO2	0.86 (0.68, 1.09)	0.21	0.58
HR	1.16 (1.10, 1.23)	<0.001	0.64
VIS	9.14 (6.18, 13.52)	<0.001	0.71
SpO2	0.80 (0.70, 0.90)	<0.001	0.58
rSO2c	0.97 (0.93, 1.01)	0.25	0.53
rSO2s	1.04 (0.999, 1.096)	0.05	0.55
SaO2-rSO2c difference	1.31 (1.11, 1.53)	0.001	0.61
SaO2-rSO2s difference	1.40 (1.21, 1.60)	<0.001	0.64

*All units of change are 10 except HR, DBP, SpO2, rSO2c, and rSO2s which are 5*.

### Multivariable Analysis

**Figure 1:**
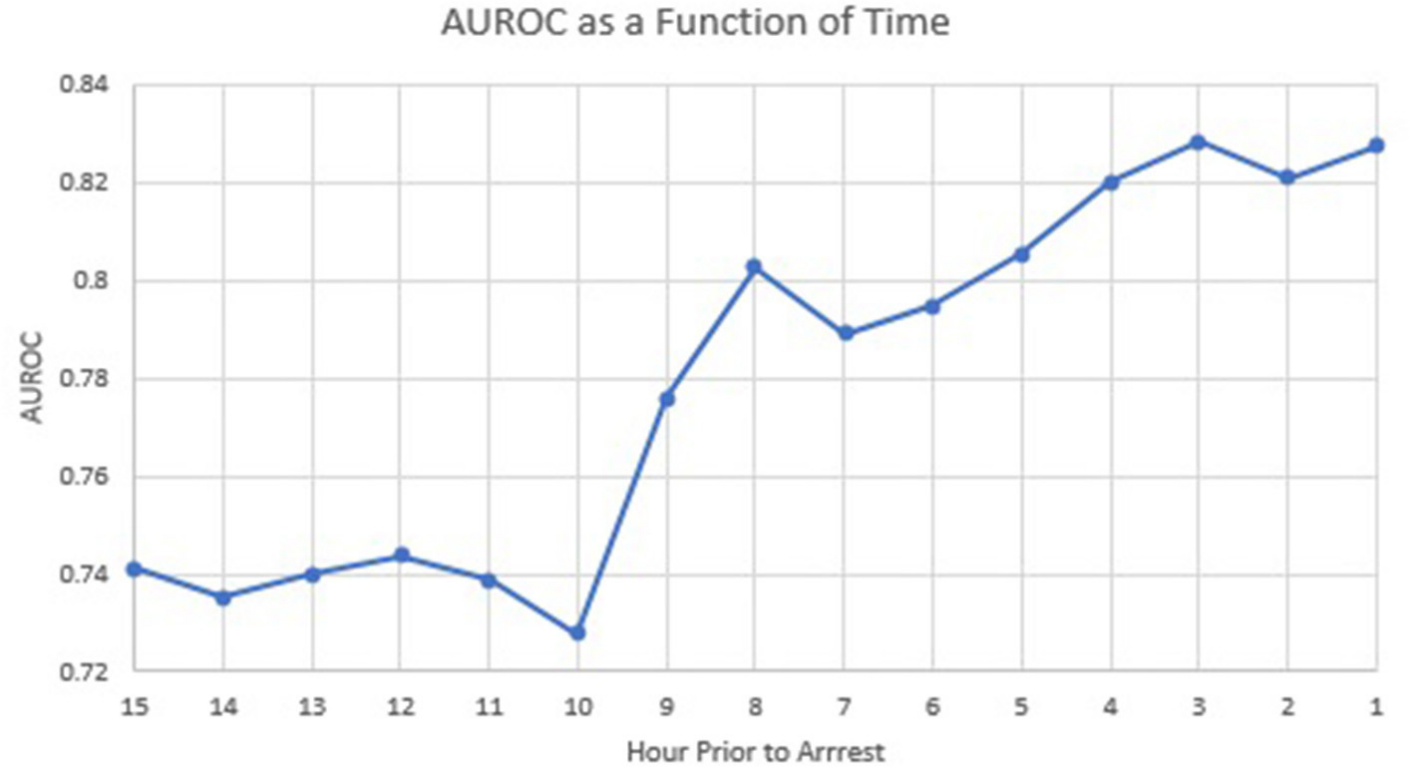
Multivariable model prediction of cardiac arrest.

Based on the univariable analysis results, we created a multivariable model consisting of FiO2, DBP, anion gap, HR, VIS, SpO2-rSO2c difference, and SpO2-rSO2s difference. Table 5 shows which variables were associated with CA with a multivariable logistic regression model. All the variables except FiO2, anion gap, and SpO2-rSO2c difference were significantly associated with CA. FiO2 and anion gap were removed from the model given their non-significance and low numbers (only 26 patients in the CA group had those variables to analyze). Figure 1 shows the graph of the multivariable logistic model with the AUROC at each hour before CA using the variables HR, VIS, SpO2-rSO2s difference, SpO2-rSO2c difference, and DBP. The AUROC increased as time moved closer to the CA and was 0.83 1 h before the CA. Table 6 displays the odds ratios for CA for each variable in the multivariable model 1 h before CA. The SpO2-rSO2s difference was independently associated with the risk of CA with an OR of 1.40 (1.18, 1.67), *P* < 0.001. The odds ratios for CA for all other hours before CA can be found in Supplementary Table 2.

**Table 5:** Variables associated with cardiac arrest in multivariable logistic regression model.

**SaO2-rSO2s difference**
HR
VIS
DBP

**Table 6:** Odds ratio of cardiac arrest of individual variables of multivariate model at 1 h prior to cardiac arrest.

**Variable**	**OR [CI]**	***P*-value**
SaO2-rSO2s difference	1.40 (1.18, 1.67)	<0.001
SaO2-rSO2c difference	1.12 (0.90, 1.38)	0.30
HR	1.17 (1.08, 1.27)	<0.001
VIS	10.01 (6.12, 16.36)	<0.001
DBP	0.95 (0.90, 0.997)	0.037

*Units of changes for HR are 5 bpm; Units of change for DBP are 5 mm Hg; SaO2-rSO2c and SaO2-rSO2s are 10% points*.

### Changes in Individual Variables in the Multivariable Model Over Time

**Figure 2:**
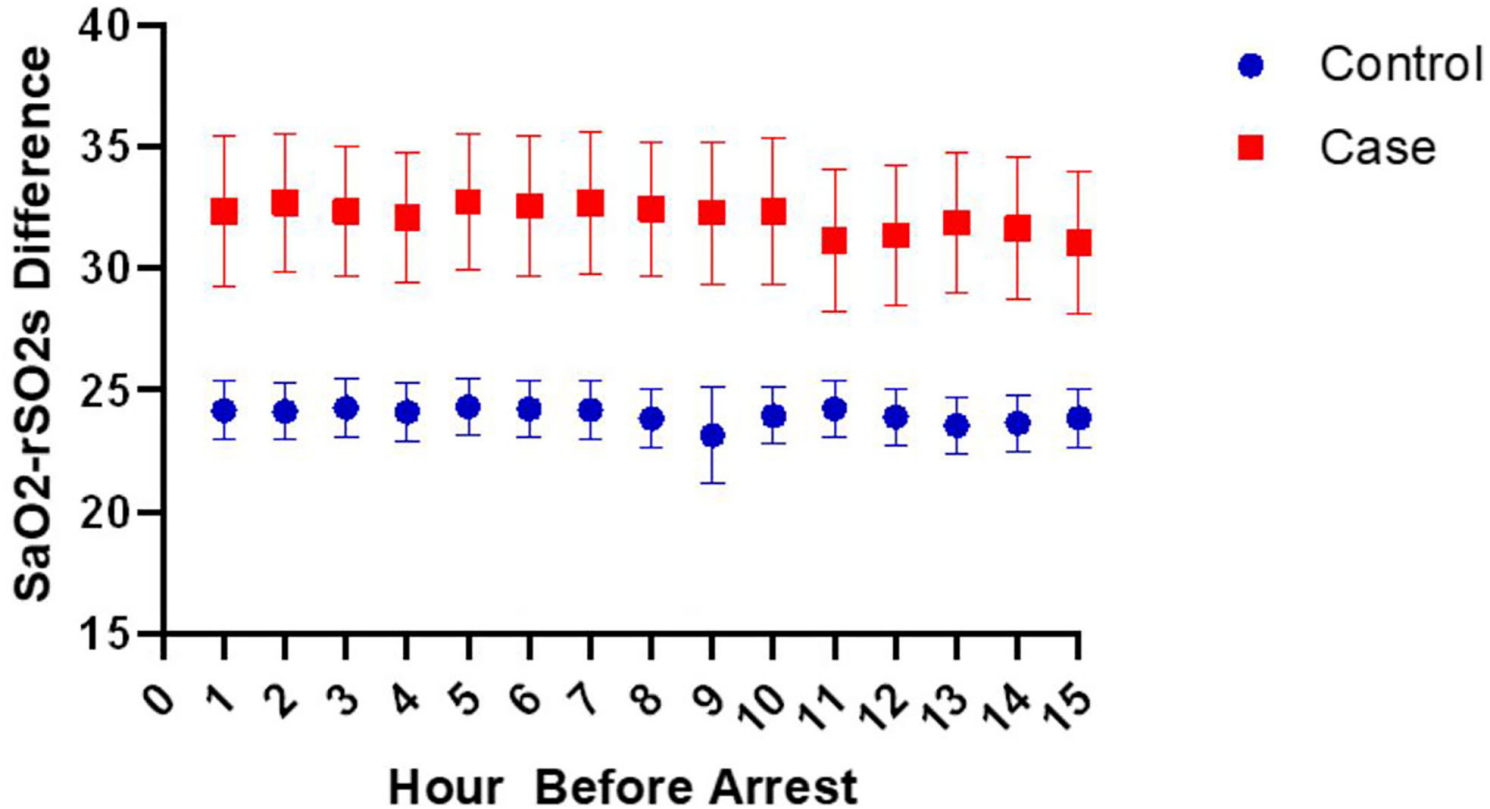
Changes in the SaO2-rSO2s difference over time.

**Figure 3:**
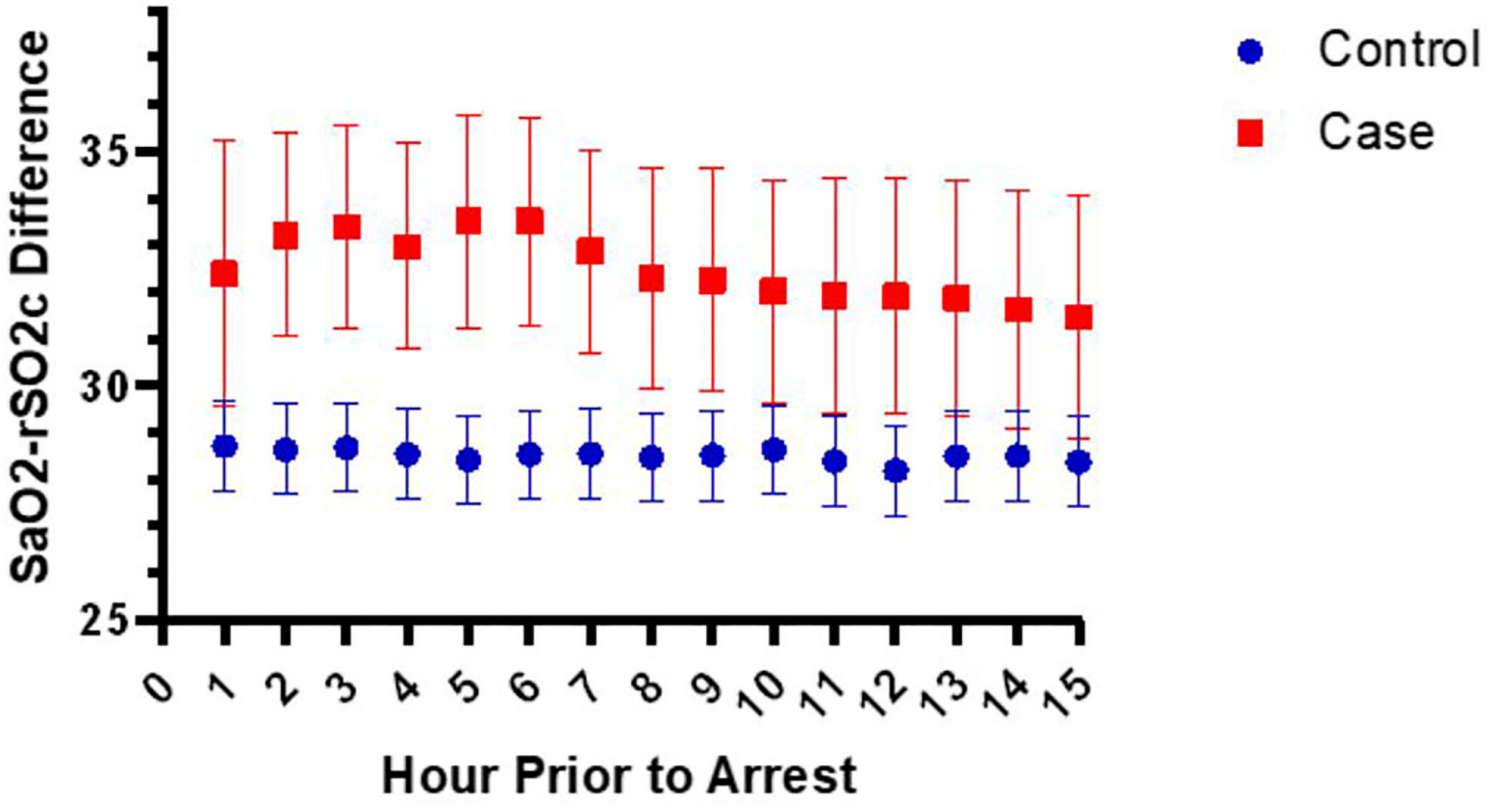
Changes in the SaO2-rSO2c difference over time.

**Figure 4:**
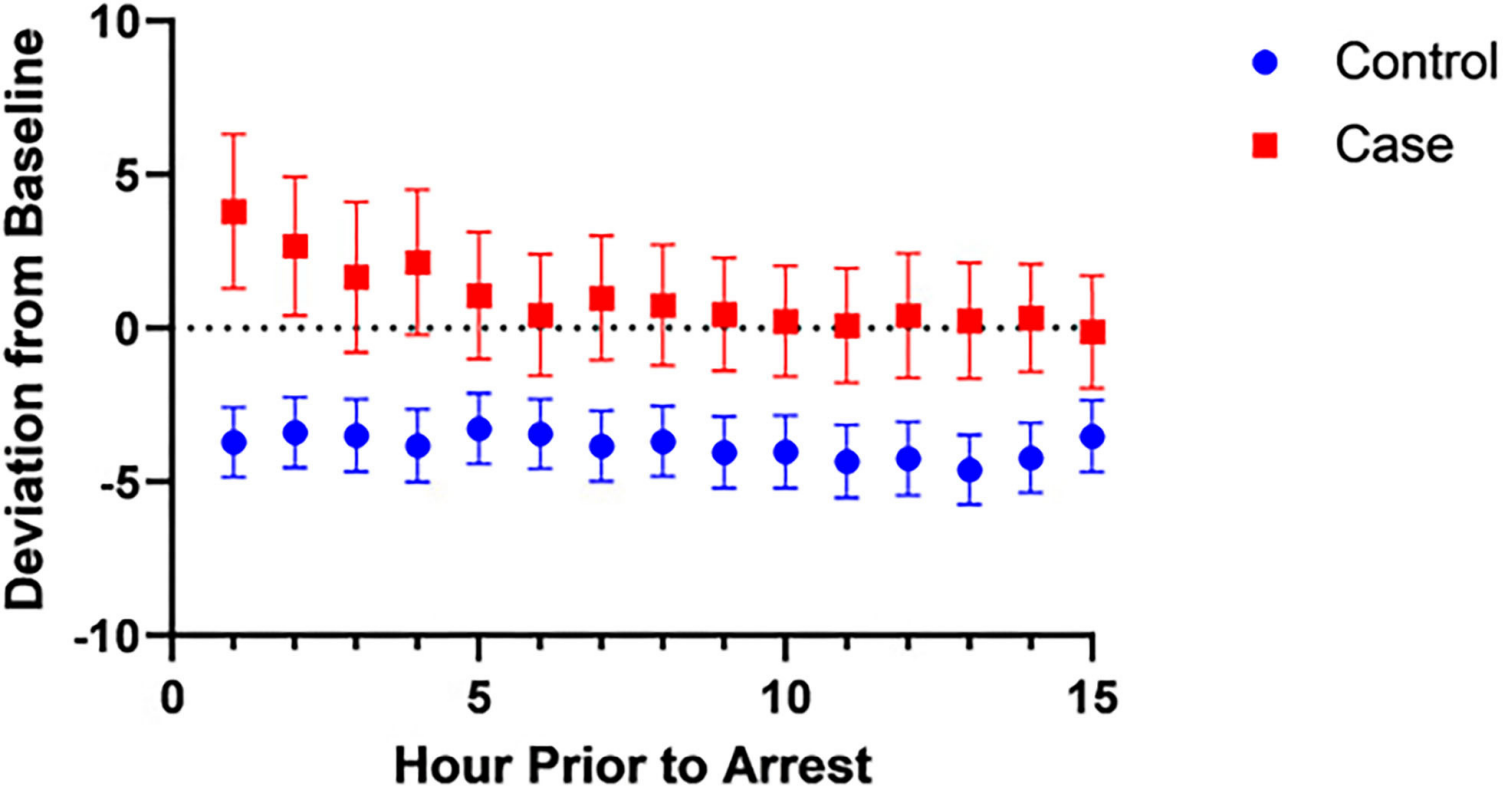
Changes in heart rate over time.

**Figure 5:**
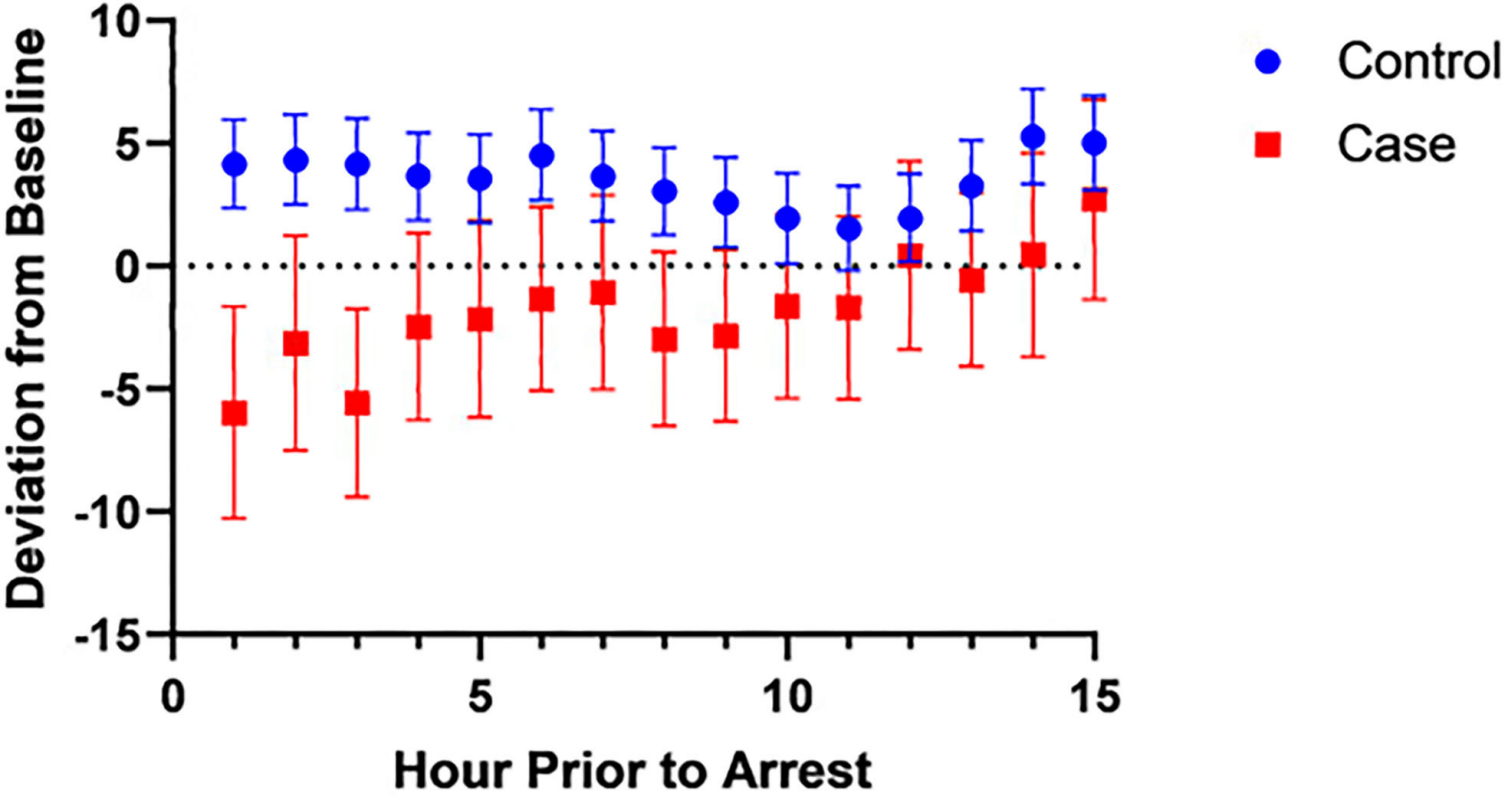
Changes in diastolic blood pressure over time.

Since the rSO2s values are affected by SpO2 values, we wanted to evaluate if it was the SpO2-rSO2s difference that was different between cases and controls, since this was taken to be a surrogate of arteriovenous oxygen difference and thus the amount of oxygen extraction. Figures 2, 3 show the mean SpO2- rSO2s difference and SpO2-rSO2c difference, respectively, over time. The SpO2-rSO2s difference values in cases were higher than in controls. The average optimal cutoff which differentiated cases and controls between hours 1 and 15 before CA was 29% [26–32%]. The SpO2-rSO2c difference values were higher in cases compared to controls as well. The average optimal cutoff which differentiated cases and controls between hours 1 and 15 before CA was 31% [30–33%]. Figures 4, 5 show changes in HR and DBP, respectively, over time. Of note, the control group's HR and DBP do not start at baseline at hour 15. By definition, we chose the baseline HR and DBP to be calculated using an average of the first 4 h of data, which for the majority of patients was before hour 15. Figure 4 shows that the control group's HR started at a higher baseline and by hour 15 came down significantly, whereas the arrest group's HR had levels at hour 15 which was similar to their baseline. Figure 5 shows that the control group's DBP baseline was similar to levels seen at hour 15, whereas the arrest group's DBP at hour 15 was lower than their baseline. At around hour 5 before CA, changes in HR and DBP start to develop. HR shows a steady increase above baseline. DBP shows a steady decline below the baseline.

### Confounding Variables

Since we were unable to match cases to controls as described previously in our Methods section, we attempted to account for confounding variables in our multivariable analysis.

The patients who had a CA compared to controls were more likely to have SV physiology, therefore, we wanted to evaluate if the diagnosis of SV physiology was a potential confounder. We also created a multivariable model that included the diagnosis of SV physiology as a covariate in addition to the other variables. Even after controlling for SV physiology diagnosis, the variable SpO2-rSO2s difference remained a significant risk factor for odds of CA.

The surgical patients who had a CA compared to the controls had higher STAT categories, longer CPB times, and longer X clamp times, therefore, we created a model for the surgical patients that included CPB times as a variable in addition to the other 4 variables. We had originally attempted to use the STAT category as a variable in our multivariable model however there was an imbalance of STAT categories in this cohort with a larger proportion of lower STAT categories. Therefore, in the multivariable analysis, we had to divide the STAT categories into two groups: those with STAT categories 1–2 vs. 3–5. Since we did not believe a dichotomous grouping adequately stratified the risk of surgical complexity, we decided to use CPB time (a variable that could be evaluated as a continuous variable) as a surrogate for surgical complexity since those patients with surgeries of higher surgical complexity tended to have higher CPB times. The CPB time was classified into 3 categories: Group 1: 0–60 min, Group 2: >60–120 min, and Group 3: >120 min. This model shows that even when controlling for CPB time, increases in the SpO2-rSO2s difference increase the odds of CA.

## Discussion

In this case-control study of children admitted to a single-center pediatric CICU, FiO2, DBP, anion gap, HR, VIS, SpO2, SpO2-rSO2c difference, and SpO2-rSO2s difference were all significantly associated with the risk of CA on univariable analysis. The multivariable analysis found that the variables HR, VIS, SpO2-rSO2s difference, and DBP were independently associated with CA. Multivariable logistic regression model to predict CA with the variables HR, VIS, SpO2-rSO2s difference, SpO2-rSO2c difference, and DBP had a good performance of an AUROC that improved over time with the highest AUROC at hour 1 before arrest of 0.83 (Figure 1). When looking at the individual components of our model over time, the SpO2-rSO2s difference and SpO2-rSO2c difference between case and control patients remained relatively constant (Figures 2, 3), whereas the HR and DBP showed steady increases and decreases, respectively, as the time approached the CA (Figures 4, 5). Interestingly, changes in HR and DBP showed similar changes at around hour 5 before CA. For the variable DBP, control patients started off and remained with values close to their baselines with CA patients having DBP values that were lower than their baseline values, but at hour 5 before CA, the split between case and control patients becomes more prominent with case patients having declines in their DBP. In contrast, with the variable HR, CA patients started with their baseline HR, while control patients started with significantly lower HR values than their baseline. Again, at hour 5 before CA, the split between the two groups becomes more prominent with case patients having HR values that increase.

Studies have shown there is usually a widening of the arteriovenous oxygen (AVO2) difference in settings of shock or poor oxygen delivery to the body. Thus, the decision to choose to use the variables SpO2-rSO2s and SpO2-rSO2c in our model. Although in our multivariable model changes in SpO2-rSO2c difference were not significant for CA, changes in SpO2-rSO2s were. Typically, when cardiac output is limited, somatic perfusion is limited to preserve cerebral perfusion (20). Therefore, when cerebral perfusion is impaired, it is often a late sign of impaired cardiac output when the body has lost its compensatory mechanism to preserve cerebral blood flow. A study by Hanson et al. showed that in moderately dehydrated children, rSO2c is preserved while rSO2s often decreases. Rehydration resulted in a significant increase in rSO2s with no changes in rSO2c (21). We only analyzed the performance of the model up to 1 h before CA. It is possible if we had evaluated rSO2c in the minutes preceding CA, we could have detected changes.

As expected, the cases had overall higher SpO2-rSO2s values compared to controls. The average optimal cutoff which differentiated cases and controls between hours 1 and 15 before CA was 29%. This optimal cutoff can be valuable for the provider taking care of children with CHD when used in conjunction with other patient factors. It was unexpected that there were no changes in SpO2-rSO2s difference closer to the arrest of cases compared to controls. We hypothesize that this could be secondary to clinician intervention. A clinician may notice early on a widened SpO2-rSO2s difference and make interventions to correct what is perceived to be a state of impending deterioration, such as administration of fluid, packed red blood cell (prbc) transfusion, or titration of vasoactive infusions. We did not investigate the administration of any medications for this study other than vasoactive infusions. Future studies should evaluate other factors that could affect SpO2-rSO2s differences, such as administration of packed red blood cell transfusions, volume expanders, sedative medications, and neuromuscular blockade. Why those patients despite stable SpO2-rSO2s differences continued to go on to have a CA is unknown. Some patients will have a CA that is unpredictable, such as from a sudden respiratory arrest from a mucus plug occluding an airway. There are other subgroups of cardiac patients who are at high risk of sudden cardiac arrest without any preceding changes, such as single ventricle physiology patients, those with coronary artery abnormalities, or primary arrhythmias. Data from the Single Ventricle Reconstruction Trial have shown that 18% of deaths during hospitalization post the Norwood procedure were sudden and unexpected (22). We did not do subgroup analysis on patients who were found to have a non-sudden CA, but perhaps future studies could delve into that subset of patients who are not thought to have a sudden event, and evaluate the changes in their SpO2-rSO2s difference.

To our knowledge, this is the first case-control study to show that a multivariable model using NIRS can be used to predict CA. There have been various case reports and case series reporting the use of NIRS as a predictor of CA. Mebius et al. published a case report of two infants with CHD who demonstrated a change in NIRS before the onset of CA (15). Tume et al. published a case report of an infant after cardiac surgery who demonstrated a decline in NIRS before the onset of CA (14). Lanks et al. published a case report of an adult who demonstrated a decrease in his cerebral NIRS before the onset of CA (10).

Although not specific to CA, prior studies have shown an association between NIRS and mortality in children with CHD which is consistent with the findings of our study. Hoffman et al. found that the use of rSO2c and rSO2s in the post-operative period could predict mortality and ECMO use in patients with hypoplastic left heart syndrome (6). Phelps et al. found that low rSO2c in the first 48 h after the Norwood procedure had a strong association with adverse outcomes, defined as hospital death, need for ECMO, or CICU stays >30 days (23).

We chose to only analyze data from the EHR rather than continuous physiologic data. It is very likely that with more granular data, our model performance would improve. Although not quite ready for bedside use, the goal would be to program a prediction model into our existing EHR to flag clinicians to patients with concerning trends. Our next step will be to use continuous physiologic data to see if this improves our model performance.

### Limitations

One of the main limitations of our study is that we only chose to study the first 48 h of the ICU admission of control patients. This would likely give us a skewed sample of patients that would not include higher post-operative stat categories from neonatal surgical repairs, such as the Norwood procedure, since it is not our center's norm to do these procedures within the first 48 h of admission given the elevated PVR and transitional physiology. The bigger changes in VIS, HR, DBP, and SpO2-rSO2s difference could be reflective of more complex surgeries and longer surgical times in patients with higher stat categories rather than a marker of impending cardiac arrest. We chose the first 48 h of admission for control patients to compare to the CA patients since we believed that for most patients (whether it was post-surgical or a medical admission), the first 48 h of admission tend to be their most unstable.

The prearrest characteristics were different in the case vs. control patients. Currently, there is no severity of illness score that has been validated in both the surgical and non-surgical pediatric heart disease population that would allow us to match cases with control patients. We believe that the large sample size of our control group will make up for this limitation. We were unable to include stat category as a covariate in our model since there was a relatively smaller number of patients with higher STAT category operations. Instead, we used CPB time as a covariate since usually operations with higher STAT categories have higher CPB times. We elected to use CPB time as a surrogate for the STAT category.

This is also a single-center study, therefore, our results may not apply to other centers. This is also a heterogeneous group of patients with various age ranges, cardiac anatomy, and physiology.

## Conclusion

In this case-control study of children admitted to a single-center pediatric CICU, we were able to show that a multivariable model consisting of SpO2-rSO2s difference, and SpO2-rSO2c difference, HR, DBP, and VIS was able to predict CA with an AUROC of 0.83 1 h to the CA. Furthermore, at 1 h before CA, for every 10% increase in the SpO2-rSO2s difference, the odds of cardiac arrest increased by 40%. The average optimal cutoff which differentiated cases and controls was 29%. Future studies should validate this model using continuous physiologic rather than hourly data to see if the model performance would be enhanced.

## Data Availability Statement

The original contributions presented in the study are included in the article/Supplementary Material, further inquiries can be directed to the corresponding author/s.

## Ethics Statement

Ethical review and approval was not required for the study on human participants in accordance with the local legislation and institutional requirements. Written informed consent from the participants' legal guardian/next of kin was not required to participate in this study in accordance with the national legislation and the institutional requirements.

## Author Contributions

PY, IE, and LR contributed to the conception and design of the study. XL performed the statistical analysis. PY wrote the first draft of the manuscript. All authors contributed to manuscript revision, read, and approved the submitted version.

## Funding

The research reported in this publication was supported by Children's Health^SM^ and the ZOLL Foundation.

## Author Disclaimer

The content is solely the responsibility of the authors and does not necessarily represent the official views of Children's Health^SM^ or the ZOLL Foundation.

## Conflict of Interest

The authors declare that the research was conducted in the absence of any commercial or financial relationships that could be construed as a potential conflict of interest.

## Publisher's Note

All claims expressed in this article are solely those of the authors and do not necessarily represent those of their affiliated organizations, or those of the publisher, the editors and the reviewers. Any product that may be evaluated in this article, or claim that may be made by its manufacturer, is not guaranteed or endorsed by the publisher.
