# Supplementary material for: Early Changes in Near-Infrared Spectroscopy Are Associated With Cardiac Arrest in Children With Congenital Heart Disease
Source: Front Pediatr. 2022 Jun 27;10:894125. doi: 10.3389/fped.2022.894125 (PMC9271890; doi:10.3389/fped.2022.894125)
Supplement: Supplementary file 1 [file Table_1.pdf]

Variable	Percentage of missing data points
SpO2-rSO2c difference	67.2%
SpO2-rSO2s difference	69.8%
Anion gap	98.5%
Base excess	92.4%
DBP	46.0%
HR	36.2%
Fio2	94.0%
rSO2c	52.9%
rSO2s	56.3%
SpO2	44.4%
ETCO2	81.6%
Urine output	36.0%
VIS	0.0%

Supplemental Table 1: Percentage of Missing Data Points by Variable
