# Supplementary material for: Early Changes in Near-Infrared Spectroscopy Are Associated With Cardiac Arrest in Children With Congenital Heart Disease
Source: Front Pediatr. 2022 Jun 27;10:894125. doi: 10.3389/fped.2022.894125 (PMC9271890; doi:10.3389/fped.2022.894125)
Supplement: Supplementary file 2 [file Table_2.pdf]

	Hour 1		Hour 2		Hour 3		Hour 4		Hour 5		Hour 6		Hour 7		Hour 8	
Variable	OR [CI]	P value	OR [CI]	P value	OR [CI]	P value	OR [CI]	P value	OR [CI]	P value	OR [CI]	P value	OR [CI]	P value	OR [CI]	P value
SaO2-rSO2s difference	1.40(1.18,1.67)	<0.001	1.34 (1.13, 1.58)	<0.001	1.42 (1.18, 1.69)	<0.001	1.31 (1.10, 1.55)	0.002	1.33 (1.12, 1.58)	0.001	1.45 (1.23, 1.72)	<0.001	1.48 (1.25, 1.75)	<0.001	1.50 (1.27, 1.78)	<0.001
SaO2-rSO2c difference	1.12(0.90,1.38)	0.30	1.12 (0.91, 1.37)	0.25	1.09 (0.89, 1.35)	0.40	1.29 (1.04, 1.59)	0.029	1.22 (0.98, 1.52)	0.063	1.08 (0.88, 1.33)	0.46	1.11 (0.9, 1.36)	0.33	1.09 (0.89, 1.33)	0.421
HR	1.17(1.08,1.27)	<0.001	1.13 (1.07, 1.36)	<0.001	1.12 (1.03, 1.21)	0.005	1.12 (1.04, 1.21)	0.003	1.16 (1.02, 1.19)	0.016	1.10 (1.01, 1.19)	0.02	1.11 (1.02, 1.19)	0.01	1.11 (1.03, 1.20)	0.008
DBP	10.01(6.12,16.36)	<0.001	0.95 (0.90, 0.998)	0.044	0.92 (0.88, 0.97)	<0.001	0.96 (0.91, 1.01)	0.13	0.97 (0.93, 1.02)	0.24	0.96 (0.91, 1.01)	0.09	0.95(0.91, 1.01)	0.079	0.93 (0.88, 0.98)	0.008
VIS	0.95(0.90,0.997)	0.037	9.29 (5.76, 14.97)	<0.001	8.56 (5.29, 13.85)	<0.001	8.25 (5.010, 13.33)	<0.001	7.39(4.61, 11.85)	<0.001	7.00 (4.38, 11.16)	<0.001	5.66 (3.58, 8.97)	<0.001	5.58 (3.52, 8.84)	<0.001

	Hour 9		Hour 10		Hour 11		Hour 12		Hour 13		Hour 14		Hour 15	
Variable	OR [CI]	P value	OR [CI]	P value	OR [CI]	P value	OR [CI]	P value	OR [CI]	P value	OR (CI)	P value	OR (CI)	P value
SaO2-rSO2s difference	1.48 (1.25, 1.75)	<0.001	1.28 (1.09, 1.5)	0.002	1.35 (1.15, 1.59)	<0.001	1.47 (1.24, 1.73)	<0.001	1.37(1.16,1.61)	<0.001	1.35(1.15,1.58)	<0.001	1.39(1.18,1.64)	<0.001
SaO2-rSO2c difference	1.03 (0.84, 1.26)	0.79	1.13 (0.93, 1.38)	0.190	1.14 (0.94, 1.38)	0.178	1.08 (0.89, 1.32)	0.43	1.16(0.96,1.42)	0.122	1.14(0.94,1.39)	0.181	1.25(1.02,1.53)	0.03
HR	1.13 (1.05, 1.22)	<0.001	1.10 (1.02, 1.19)	0.013	1.10 (1.02, 1.18)	0.018	1.11(1.04, 1.20)	0.002	1.13(1.05,1.22)	0.001	1.16(1.07,1.25)	<0.001	1.13(1.04,1.22)	<0.001
DBP	0.93 (0.88, 0.98)	0.008	0.98 (0.93, 1.03)	0.215	0.99 (0.94, 1.04)	0.67	1.009(0.96,1.05)	0.699	0.98(0.93,1.02)	0.367	0.970(0.93,1.01)	0.177	0.97(0.926,1.01)	0.18
VIS	4.16 (2.64, 6.54)	<0.001	3.45 (2.22, 5.38)	<0.001	3.26 (2.09, 5.08)	<0.001	3.09(1.98, 4.84)	<0.001	2.74(1.76,4.29)	<0.001	2.87(1.83,4.49)	<0.001	2.78(1.78,4.39)	<0.001

Units of changes for HR are 5 bpm; Units of change for DBP are 5 mm Hg; SaO2-rSO2c difference and SaO2-rSO2s difference are 10% points

Supplemental Table 2: Odds Ratio of CA for Various Models at Hours 1 through 15 prior to CA
